# Exogenous application of mycotoxin fusaric acid improve the morphological, cytogenetic, biochemical and anatomical parameters in salt (NaCl) stressed *Allium cepa* L.

**DOI:** 10.1038/s41598-024-57762-z

**Published:** 2024-03-23

**Authors:** Kürşat Çavuşoğlu, Dilek Çavuşoğlu

**Affiliations:** 1 Department of Biology, Faculty of Engineering and Natural Sciences, Süleyman Demirel University, 32260 Isparta, Turkey; 2 Department of Plant and Animal Production, Atabey Vocational High School, Isparta University of Applied Sciences, 32670 Isparta, Turkey

**Keywords:** Antioxidant enzyme, Fusaric acid, Germination, Lipid peroxidation, Mitosis, Salinity, Chromosomes, Cytogenetics, Salt, Root apical meristem

## Abstract

Salinity is one of the most important abiotic stress factors that negatively affect plant growth and development. In contrast, fusaric acid (FA), a mycotoxin produced by *Fusarium* and *Giberella* fungal genera, has biological and metabolic effects in various plants. In this study, it was aimed to investigate the protective effect of externally applied FA (0.1 nM) against the damage caused by salt (0.15 M NaCl) stress in onion (*Allium cepa* L.) plant. Salt stress resulted in an increase in the chromosomal aberrations (CAs) and micronucleus (MN) frequency, a decrease in the mitotic index (MI), fresh weight, root number, germination percentage, and root length. It promoted CAs such as irregular mitosis, bilobulated nuclei, chromosome loss, bridge, unequal seperation of chromosome, vagrant chromosome and polar slip in root meristem cells. In addition, salt stress caused a enhancement in free proline (PR), catalase (CAT), superoxide dismutase (SOD) and malondialdehyde (MDA) contents in the roots of onion plant. Moreover, it revealed damage and changes that include the accumulation of some chemical substances such as proline and sugars in epidermis and cortex layer cells, epidermal cell injury, flattening of the cell nucleus*,* wall thickening in cortex cells, necrotic areas and indistinct transmission tissue in the anatomical structure of onion roots. On the other hand, FA application promoted bulb germination and mitotic activity, strengthened the antioxidant defense system, and reduced chromosome and anatomical structure damages. In conclusion; it has been revealed that exogenous FA application may have a positive effect on increasing the resistance of onion plants to salt stress.

## Introduction

One significant abiotic factor that is recognized as endangering plant growth and productivity is salt stress in particular^1^. A issue with salt has been present on roughly 23% of agricultural lands due to improper irrigation techniques employed in farming^2^. It is well recognized that salinity in soil negatively affects a plant’s morphological, anatomical, physiological, cytogenetic, and biochemical structures, which in turn prevents a plant from growing and developing^3,4^. Furthermore, by promoting the production of free radicals in plants, salt stress directs osmotic and oxidative stresses^5^.

However, because they have a variety of defense mechanisms, including osmoregulation, ion sequestration, up-regulation of antioxidant activities, and tolerance to salinity stress, plants can fend off osmotic and oxidative stresses^6,7^. Consequently, boosting the concentration of many non-enzymatic substances like salicylate, ascorbate, and carotenoids as well as antioxidant enzymes like SOD and CAT is crucial for tolerance to salinity^8,9^.

Plants and plant pathogens create highly harmful chemicals called phytotoxins as secondary metabolites. These compounds, which are also known as poisons or plant-allelochemicals^10^, are frequently present in organs such fruits, flowers, leaves, bark, and roots^11^. Phytotoxins are commonly utilized in agriculture and typically exhibit their stimulating effects on plants at low concentrations^12^. These toxins serve as growth-promoting agents and defensive proteins, enabling plants to tolerate a wide range of biotic and abiotic stress conditions^13^.

Fusaric acid (FA), also known as 5-butylpicolinic acid or 5-n-butyl-pyridine-2-carboxylic acid, is a well-known phytotoxin produced by many species of *Fusarium*^14^. Numerous studies have been conducted on the physico-chemical effect of FA at the subcellular level^15–17^. However, a significant part of the works focused on the toxic influences of exogenous FA in plants^18^. It alters the permeability of mitochondrion and plasma membrane^19^, increases electrolyte leakage^20^, causes modification of membrane potential^21^, decreases ATP levels in cells^22^ and blocks various metal-containing (eg cytochrome oxidase) enzymes^20^. On the other hand, Bouizgarne et al.^23^ reported that FA doses below 10^–5^ M could induce different protective responses in plant cells.

One of the largest genera of monocots is *Allium*, which has over 850 species^24^. An important cultivated plant, *Allium cepa* L. is used as a popular culinary vegetable around the world^25^. For a variety of reasons, including its ease of availability and cultivatin, low count of chromosomes and huge chromosomes, and ease of measuring biochemical processes, it is favored as a bioindicator in experimental studies^26^. The use of *Allium cepa* L. for testing purposes was first proposed in 1938. The first application was made in 1985^27,28^. The *Allium* test is used to determine mutagenicity and toxicity^29^. Toxicity is measured by observing root growth inhibition, and mutagenicity by determining CAs rates. Cytotoxicity tests using in vivo plant test systems such as *A. cepa* have been approved by many researchers working with in vitro animal organism tests, and the results have been reported to be similar^30,31^. Because this test is an alternative, reliable, short-term and cost-effective, it has been certified by the United Nations Environment Program (UNEP) and the International Chemical Safety Program (IPCS) as an effective biotest for monitoring mutagenic effects^32^.

There is no present literature data related to the effects of exogenous FA application on all parameters examinated in this study of *Allium cepa* L. In this study, it was aimed to determine the potential role of FA in increasing the salt tolerance of onion plants.

## Materials and methods

### Choosing the experimental material and the appropriate doses

This study was carried out with bulbs of onion (*Allium cepa* L.) grown as an important agricultural plant. Bulbs were purchased from a commercial store in Isparta/Turkiye. Fusaric acid/FA (CAS number: 536-69-6) and sodium chloride/NaCl (CAS number: 7647-14-5) were supplied from Merck Company. We found that the NaCl stress level to which the bulbs are subjected 0.15 M and that the externally administered FA dose is 0.1 nM based on the results of a preliminary experiment we conducted. Experimental research on plant samples, including the supply of plant material, complies with institutional, national and international guidelines and legislation.

### The growing circumstances and the experimental strategy

A selection of roughly equivalent-sized, plump, and healthy bulbs was made. Bulbs divided into four separate groups. Twenty bulbs from each identified group were put into sterile plastic 1.7-L containers with a perforated cover with the root sections inside and the remaining parts outside. The bulbs were then allowed to germinate for 168 h (7 days) in the dark in an incubator that was kept at 20 °C. First group control bulbs were kept in tap water medium until the end of the study; second group bulbs were kept in salt stress (0.15 M NaCl) medium; third group bulbs were kept in FA (0.1 nM) medium; and fourth group bulbs were germinated in FA treated medium in addition to salt stress (0.15 M NaCl + 0.1 nM FA).

The hairy roots of the germinated bulbs in the control and treatment groups were counted at the conclusion of the 168-h application period. Root lengths were measured in millimeters (mm) using a ruler fitted with millimetric scales. The obtained root length values were expressed in meters (m). Precision balance was used to determine fresh weights in grams. The germination percentages (%) of the bulbs were calculated based on the radicle protruding 10 mm (0.01 m) from the testa as the germination criterion. In this investigation, every trial was set up in triplicate to enable statistical analysis of the collected data.

### Calculating the chromosomal aberrations and the mitotic index

Materials cut to a length of 1–2 cm from the onion root tips were immersed in saturated paradichlorobenzene for 4 h, fixed in a mixture of 3 parts ethyl alcohol and 1 part acetic acid solution, and then stored in 70% ethyl alcohol in order to identify chromosomal damage. In order to prepare the root tips permanently, they were first hydrolyzed in 1 N HCl at 60 °C for 17 min, then stained for 1 to 1.5 h with Feulgen, crushed in 45% acetic acid on a slide, covered with a coverslip, balm applied around the coverslip, and photographed at 100X magnification using a microscope^33^. 30.000 cells were counted for each root tip from the prepared preparations in order to calculate the mitotic index (MI), and Eq. (1) was used to determine the percentage of cells entering mitosis. In order to calculate chromosomal abnormalities (CAs), 2.000 dividing cells were counted.

1 $${\text{MI }}\left( \% \right) = \left[ {{\text{number}}\;{\text{of}}\;{\text{cells}}\;{\text{undergoing}}\;{\text{mitosis/total}}\;{\text{number}}\;{\text{of}}\;{\text{cells }}} \right] \times 100$$

### Determination of antioxidant enzyme activity using spectrophotometry

After the end of the applications, the roots were cut and washed with distilled water and were thoroughly crushed in a mortar in liquid nitrogen. 5 mL cold sodium phosphate buffer (50 mM, pH 7.8) was used to homogenize 0.2 g of root sample. After passing through coarse filter paper, the homogenate was centrifuged for 20 min at 10.000 rpm. The activity of the enzymes catalase (CAT) and superoxide dismutase (SOD) were measured spectrophotometrically using the supernatant.

SOD activity was determined by measuring the ability of SOD to inhibit the photochemical reduction of nitro blue tetrazolium (NBT). To determine total SOD activity; 0.01 mL enzyme extract was added to the mixture of 1.5 mL sodium phosphate buffer (0.05 M, pH 7.8), 0.3 mL methionine (130 mM), 0.3 mL riboflavin (20 μM), 0.3 mL EDTA-Na_2_ (0.1 mM), 0.3 mL nitroblue tetrazolium chloride (750 μM), 0.01 mL polyvinylpyrrolidone (4%) and 0.28 mL distilled water. Following the addition of the enzyme-containing extract, the new mixture was exposed to 375 µmol m^−2^ s^−1^ fluorescent light for 15 min to allow catalysis to occur. The reaction was stopped by turning off the light source. The color bleaching intensity of NBT within 15 min was read against blank at 560 nm on a spectrophotometer^34^, and the average total SOD activity for each group was calculated as U mg^−1^ FW^35^. Blind; it consists of an enzyme-free example of the same process^34^.

CAT activity was determined by monitoring the absorbance drop at 240 nm. In 1.5 mL sodium phosphate buffer (200 mM pH 7.8), 0.3 mL 0.1 M H_2_O_2_, 0.2 mL supernatant, and 1.0 mL deionized water were incubated for two minutes at 37 °C. The reaction was then stopped with 1 M HCl. The amount of enzyme needed to break down one μmol of H_2_O_2_ was defined as one unit of enzyme activity. The expression for CAT activity was OD240nm min g^−1^ FW^36^.

### Determination of lipid peroxidation using spectrophotometry

The expression for lipid peroxidation is the concentration of malondialdehyde (MDA). After homogenizing a 0.5 g sample of onion roots with 10 ml of 5% trichloroacetic acid (TCA), the homogenate was centrifuged for 15 min at 12.000 rpm and 24 °C. One milliliter of the clear portion of the centrifuged sample was extracted, and to it was added four milliliters of 20% TCA diluted in 0.5% thiobarbituric acid (TBA). The mixture was rapidly chilled in an ice bath after being maintained at 96 °C for twenty-five minutes and centrifuged at 10.000 rpm for five minutes. Next, the absorbance from the clear portion was measured at 532 nm, and the MDA concentration was computed using the extinction coefficient of 155 M^−1^ cm^−1^ and represented as µmol^37^.

### Determination of free proline amount using spectrophotometry

A 0.5 g fresh root sample was homogenized using 10 mL of 3% sulfosalicylic acid. After that, Whatman filter paper was used to filter the root samples. A volume of 2 ml was extracted, followed by an equal volume of acid anhydrin and glacial acetic acid. The combination was stored for one hour in a water bath at 100 °C and for 5 minutes in an ice bath. To create two phases, 5 mL of toluene was added to the reaction mixture, vortexed for 15 to 20 s, and then allowed to settle. Using a micropipette, the upper phase was obtained, and the absorbance values were measured in the spectrophotometer at 520 nm in comparison to the pure toluene control. The outcomes of the L-proline standard were contrasted with the examples’ results. With the use of Eq. (2), the amount of free proline was determined, and µg g^−1^ is represented as fresh weight^38^.

2 $$\left[ {\left( {\upmu {\text{gproline}}/{\text{mL}} \times {\text{mL}}\;{\text{toluene}}} \right)/115.5\;\upmu {\text{g}}/\upmu {\text{mole}}} \right]/\left[ {\left( {{\text{g}}\;{\text{sample}}} \right)/5} \right] =\upmu {\text{molesproline}}/{\text{g}}\;{\text{of}}\;{\text{fresh}}\;{\text{weight}}\;{\text{material}}$$

### Microscopic observations in the root anatomical structure

Cross-sections were cut from the root tips of the bulbs treated with FA and NaCl for 168 h in order to determine anatomical damage. The sections were then stained with 2% methylene blue, closed using entellan, and photographed at 500X magnification^39^.

### Statistical analysis of the data obtained

Using the SPSS 23 analytical software for Windows, statistical analyses of the collected data were performed, and the variations in the outcomes were shown as mean ± standard deviation. It was examined using a one-way ANOVA and the Duncan test at the *p* < 0.05 significant level.

### Ethical approval

The authors confirm that the manuscript has been read and approved by all authors. The authors declare that this manuscript has not been published and not under consideration for publication elsewhere.

## Results and discussion

### The role of FA on the morphological parameters

**Figure 1:**
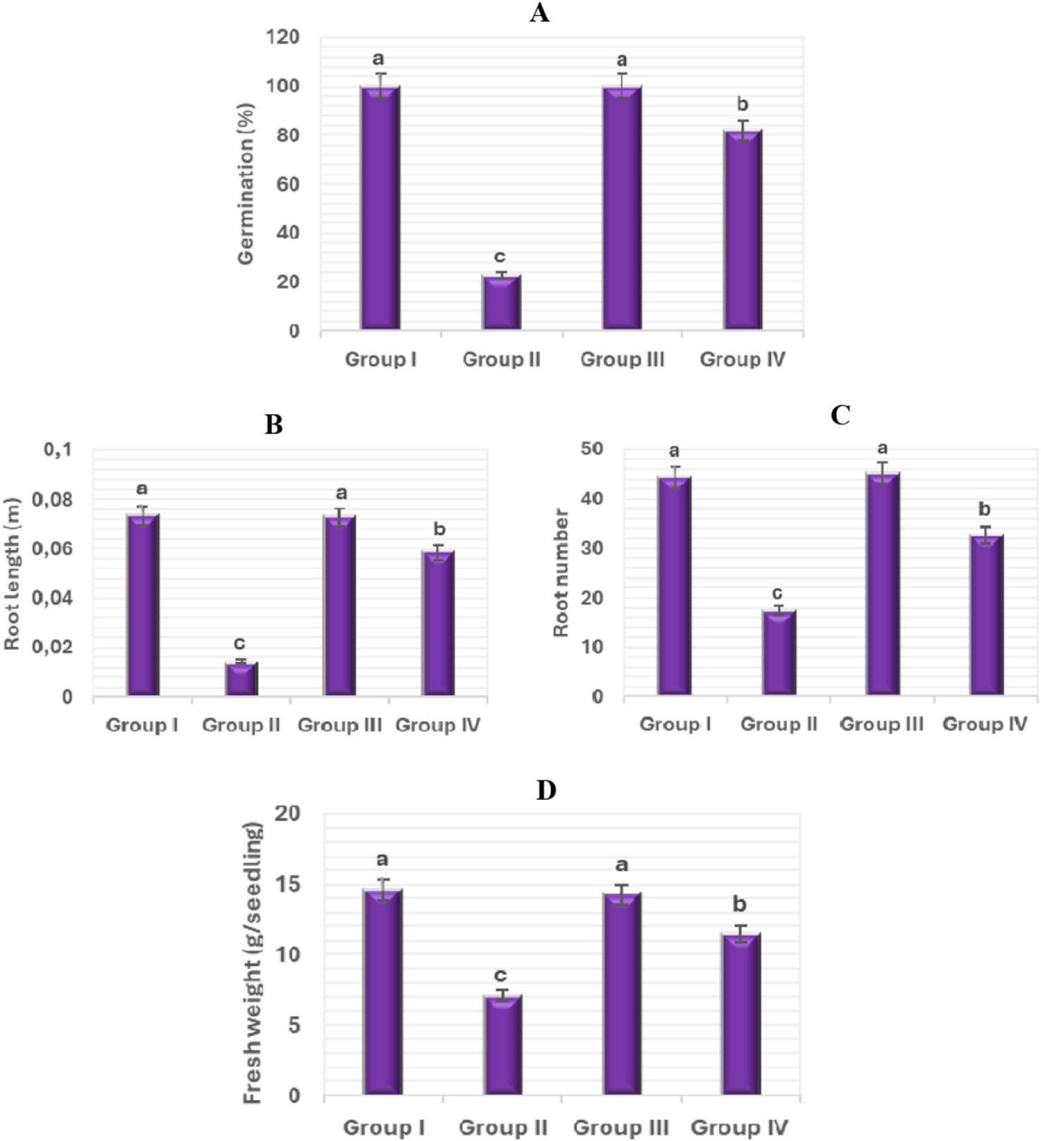
Impacts of exogenous FA administration on certain morphological parameters of *Allium cepa* bulbs. (**A**) Germination (%) (**B**) Root length (m) (**C**) Root number (**D**) Fresh weight (g/seedling). Tap water medium (control)/Group I, 0.15 M NaCl medium/Group II, 0.1 nM FA medium/Group III, 0.1 nM FA + 0.15 M NaCl medium/Group IV. Standard deviation (± SD) shown with error bars. Different letters^(a–c)^ indicate average *p* < 0.05 is important.

The effects of salt stress (0.15 M NaCl), FA (0.1 nM) and salt + FA (0.15 M NaCl + 0.1 nM FA) treatments on some morphological parameters in onion plants are given in Fig. 1. There was no statistically significant difference in germination percentage, root length, root number, and fresh weight of bulbs (Group III) treated with FA only compared to control (Group I) bulbs grown in tap water medium. The germination percentage, root length, root number, and fresh weight of Group I (control) bulbs were found to be 100 ± 0.0%, 0.0735 ± 1.2 m, 44.3 ± 1.4, and 14.6 ± 0.8 g, respectively. For Group III bulbs, these parameters were found to be 100 ± 0.0%, 0.0728 ± 2.3 m, 45.1 ± 1.9, and 14.3 ± 0.6 g. Similar results were obtained from studies with giant witchweed and watermelon plants^40,41^. However, there are studies showing that FA application alone has inhibitory^42,43^ or stimulating^44^ effects on the aforementioned parameters of giant witchweed, maize and potato plants. As a result, it can be said that FA applied externally under stress-free conditions has different effects depending on the plant species, application method and application dose.

**Figure 2:**
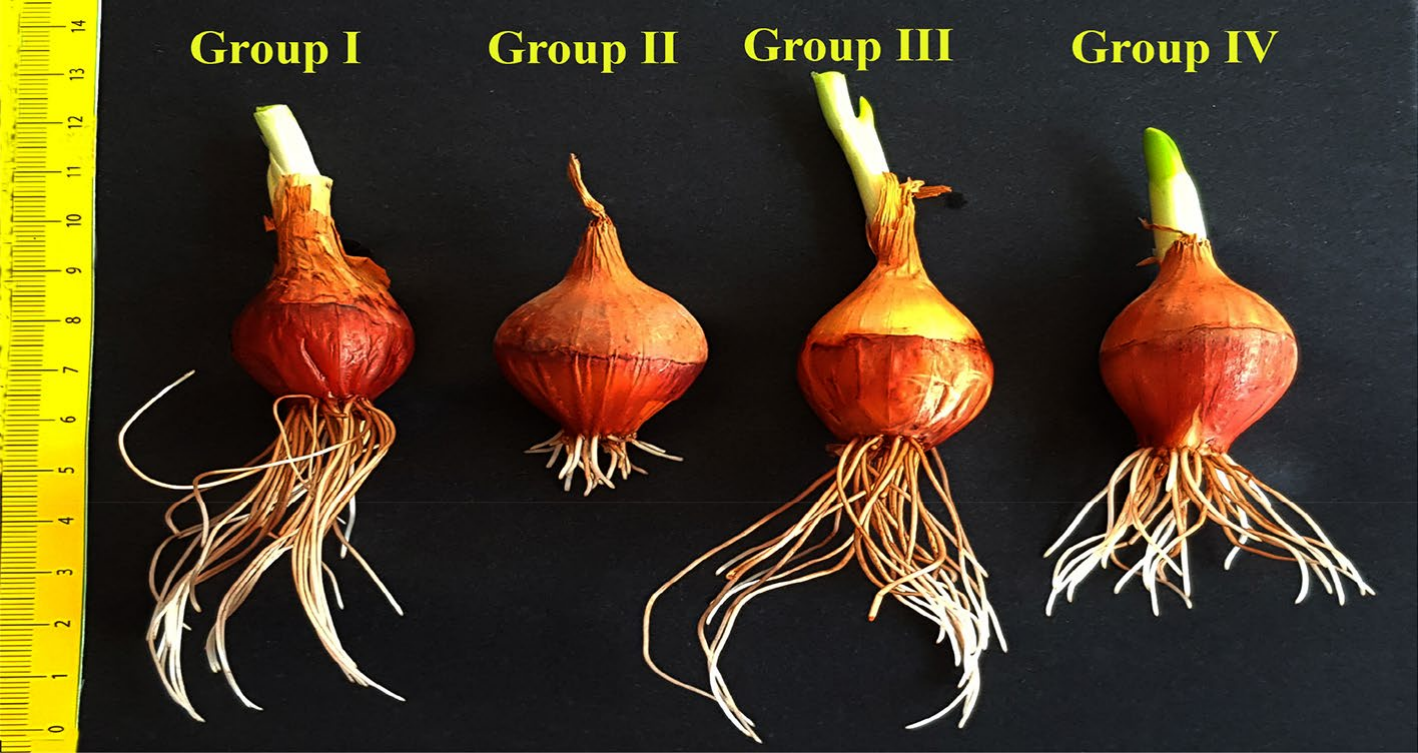
The effect of FA application on *Allium cepa* root growth under saline conditions. Tap water medium (control)/Group I, 0.15 M NaCl medium/Group II, 0.1 nM FA medium/Group III, 0.1 nM FA + 0.15 M NaCl medium/Group IV.

**Figure 3:**
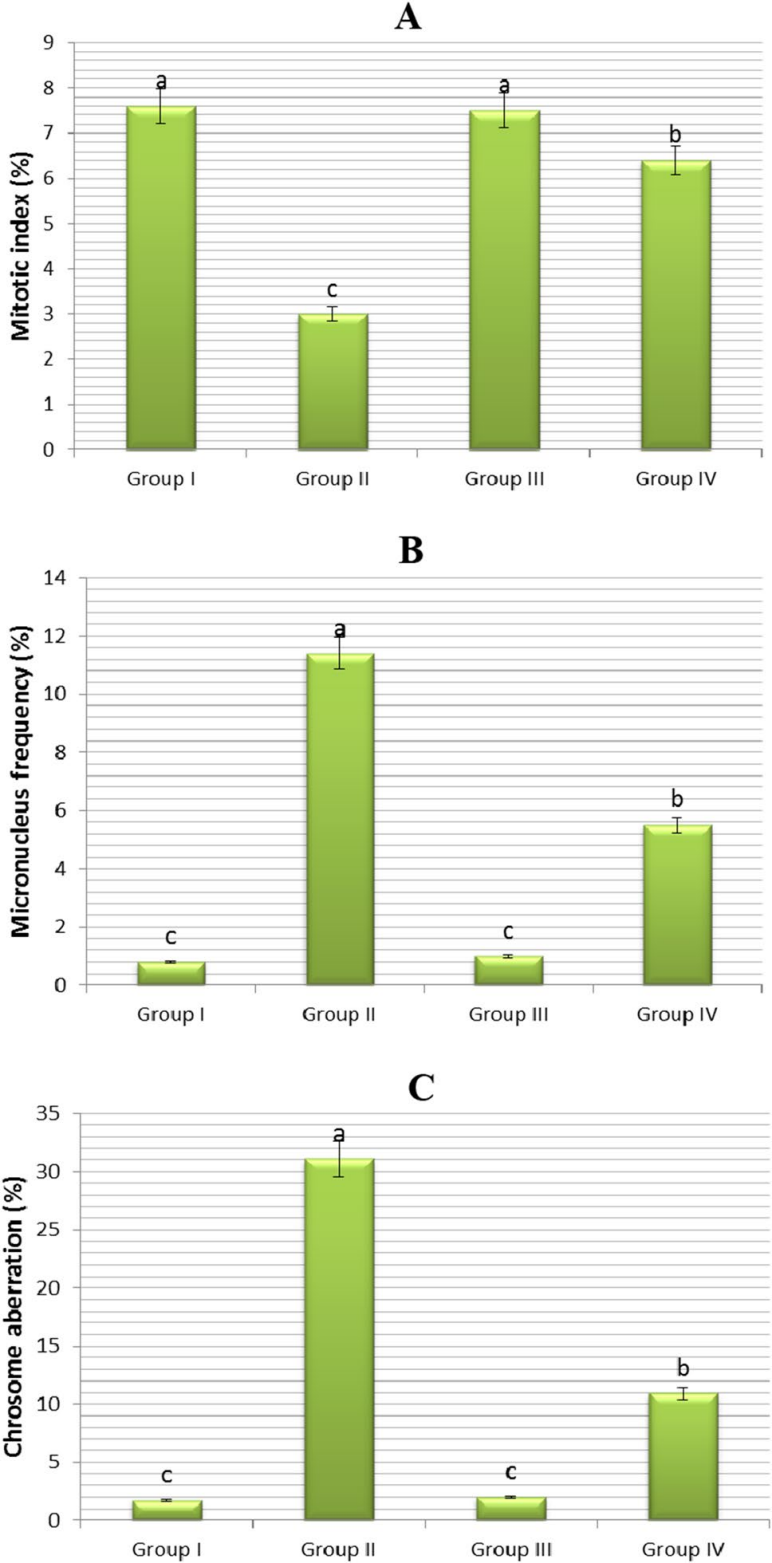
Impacts of exogenous FA administration on certain cytogenetic parameters of *Allium cepa* bulbs. (**A**) Mitotic index (%) (**B**) Micronucleus frequency (%) (**C**) Chrosome aberretion (%). Tap water medium (control)/Group I, 0.15 M NaCl medium/Group II, 0.1 nM FA medium/Group III, 0.1 nM FA + 0.15 M NaCl medium/Group IV. Standard deviation (± SD) shown with error bars. Different letters^(a–c)^ indicate average p < 0.05 is important.

It is well known that salinity causes adverse effects on bulb growth (root elongation, root number and fresh weight) and germination^45,46^. The results of this study indicated that Group I (control) bulbs grown in tap water medium had germination percentage, root length, root number, and fresh weight of 100 ± 0.0%, 0.0735 ± 1.2 m, 44.3 ± 1.4, and 14.6 ± 0.8 g, respectively; whereas these parameters showed a statistically significant decrease (p < 0.05) in Group II bulbs grown in 0.15 M NaCl medium, with values of 23 ± 2.8%, 0.0144 ± 1.2 m, 17.4 ± 0.9, and 7.1 ± 0.3 g. In other words, 0.15 M salt stress application significantly reduced the germination percentage of bulbs (Group II) by 77%, root length by 80%, root number by 61%, and fresh weight by 51% compared to control/Group I (Figs. 1 and 2). Salinity stress may have exerted its negative effects on these parameters by affecting various metabolic processes. First, because of the high osmotic pressure, the roots cannot get enough water, and the fresh weight and water content of the bulbs decreases (Fig. 1). Second, salinity inhibits mitotic activity in root tip meristem cells and the root number and length of bulbs are reduced (Fig. 3).

On the other hand, the germination percentage, root length, root number, and fresh weight parameters improved with exogenous FA treatment. Group II bulbs grown in 0.15 M NaCl medium had 23 ± 2.8% germination percentage, 0.0144 ± 1.2 m root length, 17.4 ± 0.9 root number, and 7.1 ± 0.3 g fresh weight. In FA-treated Group IV bulbs grown at this salt level, these parameters were 82.1 ± 2.8%, 0.0586 ± 1.5 m, 32.7 ± 1.1, and 11.5 ± 0.5 g, respectively. That is, exogenous FA application (0.1 nM FA + 0.15 M NaCl) in onion (Group IV) plants under salt stress increased germination percentage, root length, root number, and fresh weight by 59%, 307%, 88%, and 62%, respectively, when compared to salt-stressed plants alone (Grup II). Moreover, this application enabled the mentioned parameters to reach statistically the values obtained in control (Grup I) plants (Fig. 1).

Externally applied FA can improve the salt tolerance of the onion plant by increasing the water uptake of the roots (Fig. 1), by accelerating mitosis in the root cells (Fig. 3), by regulating the accumulation of osmolyte (like proline) substances and antioxidant (such as SOD and CAT) enzymes in the cells, and by minimizing the cell membrane damage/lipid peroxidation (Fig. 6). Thus, it can serve to a healthy germination and seedling growth. The morphological findings of the present study are very important, as there is no study on the protective role of mycotoxin FA on the germination and seedling growth in saline conditions.

### The role of FA on the cytogenetic parameters

The effects of salt stress (0.15 M NaCl), FA (0.1 nM) and salt + FA (0.15 M NaCl + 0.1 nM FA) treatments on some cytogenetic parameters in the root tip meristem cells of onion plant are given in Fig. 3. There was no statistically significant difference in mitotic index (MI), micronucleus (MN) frequency, and chromosome aberrations (CAs) in the root cells of bulbs (Group III) treated with FA only compared to control (Group I) bulbs grown in tap water medium. The MI, MN frequency, and CAs in the root cells of Group I (control) bulbs were found to be 7.6 ± 0.9, 0.8 ± 0.7, and 1.7 ± 0.6%, respectively. For Group III bulbs, these parameters were found to be 7.5 ± 0.7%, 1.0 ± 0.9%, and 2.0 ± 0.9%. In other words, FA application alone showed a statistically equivalent activity to the control (Group I) group.

This study’s findings indicated that salt stress significantly harmed chromosomal structure in addition to reducing mitotic activity. In the root tip cells of Group I (control) bulbs grown in tap water medium, MI was 7.6 ± 0.9%; in the root tip cells of Group II bulbs grown in 0.15 M NaCl medium, this value was 3.0 ± 0.2%. Moreover, the root cells of Group I (control) bulbs grown in tap water medium had 0.8 ± 0.7% MN frequency and 1.7 ± 0.6% CAs; whereas these parameters showed a statistically significant increase (*p* < 0.05) in the root cells of Group II bulbs grown in 0.15 M NaCl medium, with values of 11.4 ± 1.7% and 31.1 ± 2.2, respectively. In other words, the application of 0.15 M salt stress decreased MI approximately 2.53 times, while it increased the MN frequency by 14.25 times and CAs by 18,29 times in root tip cells of bulbs (Group II) compared to control/Group I (Fig. 3). Cytotoxicity is defined as a decrease in the MI^47^ and an increase in the fraction of cells with abnormal chromosomes^48^. It has been reported that MI decreased and CAs increased^49–51^ in root cells of plants exposed to salt stress, which is in line with the present research results. In test organisms, a decrease in the MI below 22% of the control causes a lethal effect^52^, while a decrease below 50% generally causes a sublethal effect^53^ and this value is called the cytotoxic cutoff value^54^. In this study, it was observed that salt stress had a sublethal effect. Because, the MI value was only 3.0% at 0.15 M NaCl concentration. This value was approximately 40% of the C (7.6%).

On the other hand, application of 0.1 nM exogenous FA to Group IV bulbs grown in 0.15 M NaCl medium resulted in a significant decrease in MN frequency and CAs, an increase in MI (p < 0.05). The root tip meristem cells of Group II bulbs grown in 0.15 M NaCl medium had 3.0 ± 0.2% MI, 11.4 ± 1.7% MN frequency, and 31.1 ± 2.2% CAs. In the root cells of FA-treated Group IV bulbs grown at this salt level, these parameters were 6.4 ± 0.7%, 5.5 ± 1.4, and 10.9 ± 1.6, respectively. That is, exogenous FA application (0.1 nM FA + 0.15 M NaCl) in onion (Group IV) plants under salt stress increased MI by 113%, and decreased MN frequency by 52% and CAs by 65%, when compared to salt-stressed plants alone/Grup II (Fig. 3). This study is the first study in the literature on the effects of FA on cell division and chromosome behavior in plant cells under both non-stress and salt stress conditions. Moreover, it has been demonstrated that salt stress damage on chromosomal structure and MI in onion cells can be significantly reduced by FA application.

**Figure 4:**
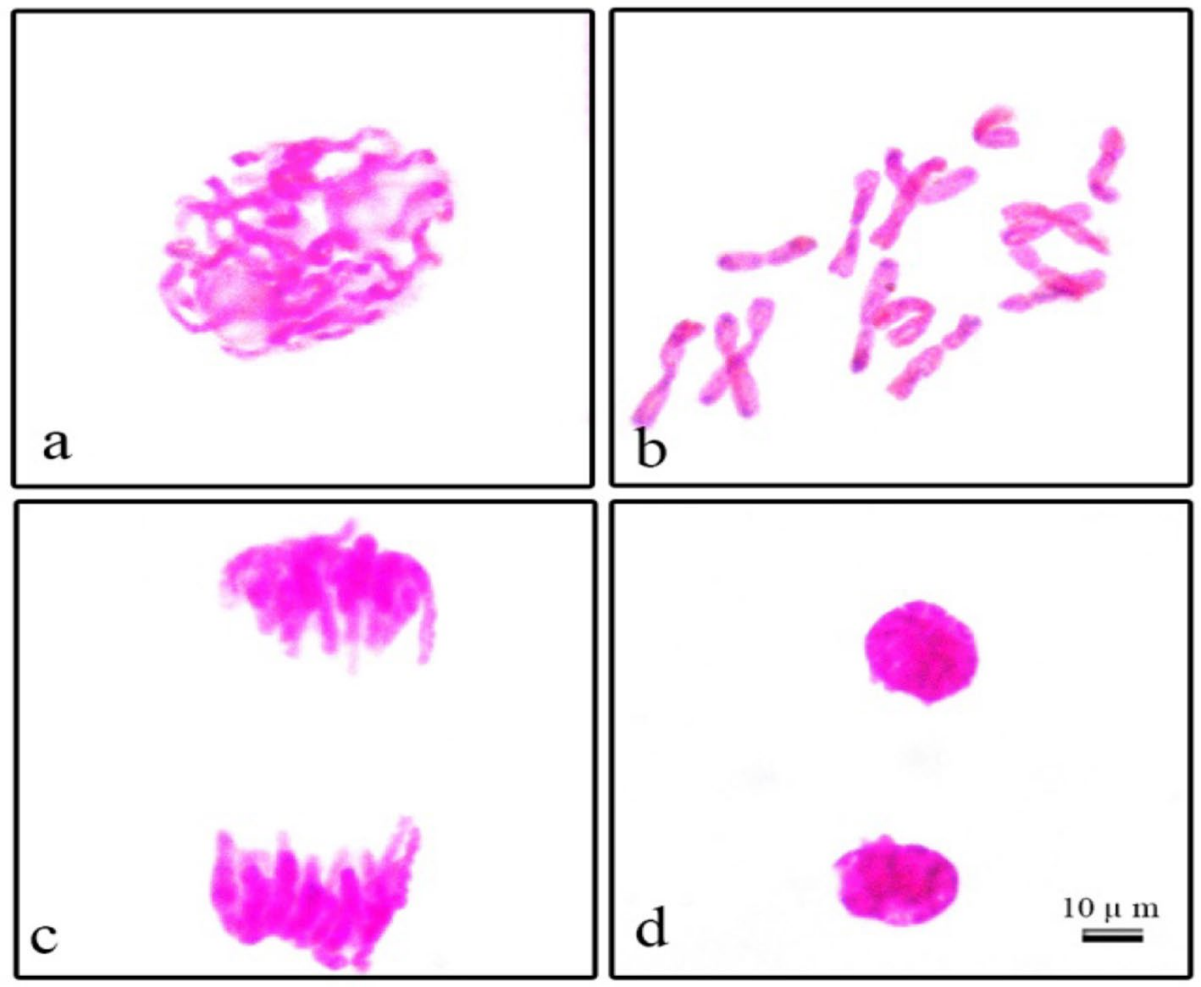
Normal mitotic phases observed in root cells of control group *Allium cepa* samples germinated in tap water environment (**a**) Prophase (**b**) Metaphase, 2n = 16 chromosomes (**c**) Anaphase (**d**) Telophase. Scale bar = 10 μm.

**Figure 5:**
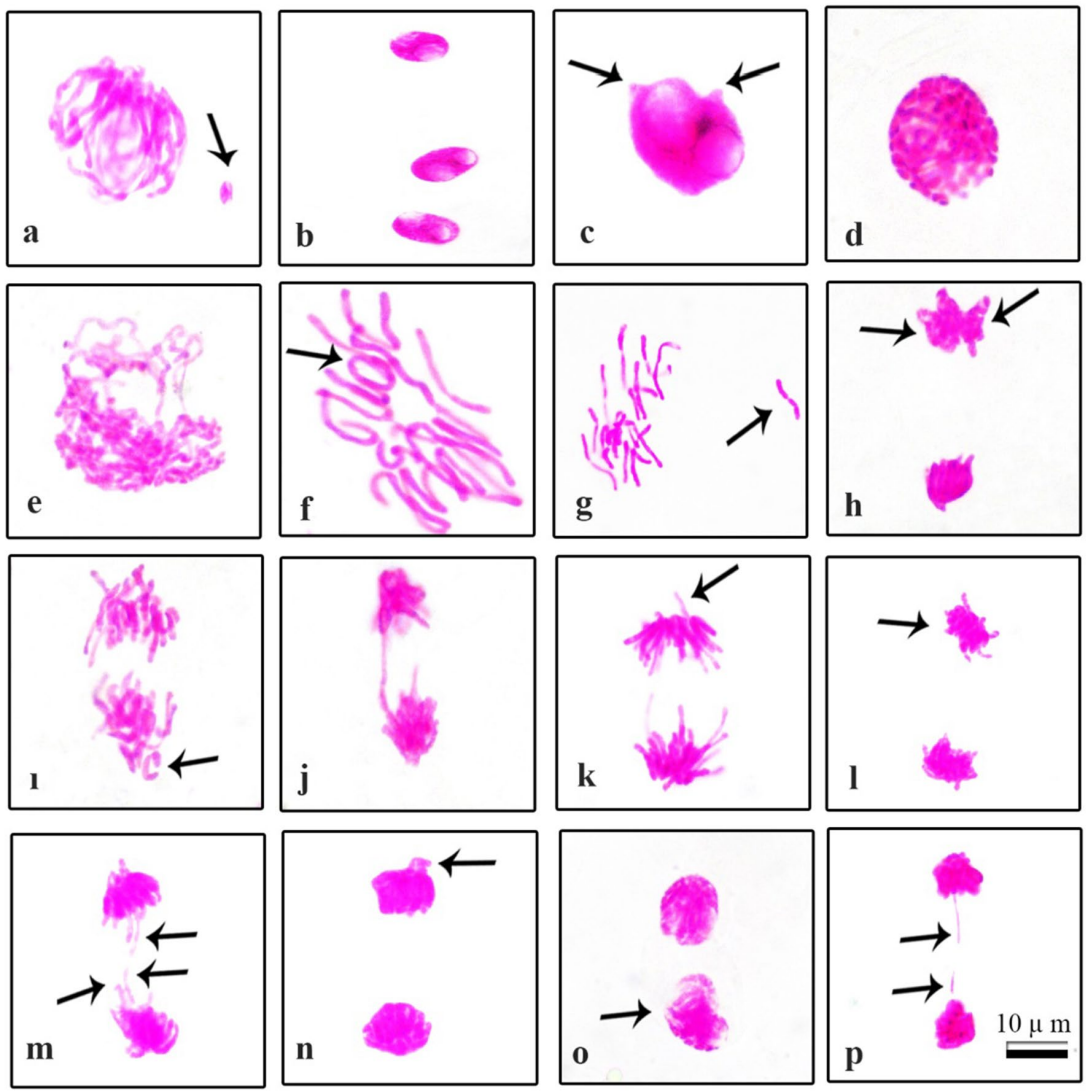
Abnormal chromosomes observed in root cells of application group *Allium cepa* specimens germinated in NaCl medium (**a**) micronucleus(= arrow) (**b**) bilobulated nuclei (**c**) bilobulated nucleus with buds(= arrow) (**d**) chained prophase (**e**) irregular prophase (**f**) metaphase with chromosome encircled(= arrow) (**g**) metaphase with chromosomal loss(= arrow) (**h**) unequal seperation of chromosome at anaphase stage(= arrows) (**i**) anaphase with chromosomal loss(= arrow) (**j**) bridge in anaphase (**k**) anaphase with vagrant chromosome(= arrow) (**l**) anaphase with polar slip(= arrow) (**m**) anaphase with lagging chromosomes(= arrows) (**n**) telophase with vagrant chromosome(= arrow) (**o**) telophase with polar slip (= arrow) (**p**) telophase with lagging chromosomes(= arrows). Scale bar = 10 μm.

As a result of the counts and microscopic examinations in the control group (Group I) onion cells, it was observed that most of the mitotic cells were in the normal chromosome structure and the chromosome number of the onion mitotic metaphase cells was determined as 2n = 16 (Fig. 4). A total of 13 types of abnormalities, including micronucleus (Fig. 5a), bilobulated nucleus (Fig. 5b, c), buds (Fig. 5c), chained prophase (Fig. 5d), irregular prophase (Fig. 5e), metaphase with chromosome encircled (Fig. 5f), metaphase with chromosomal loss (Fig. 5g), unequal seperation of chromosome at anaphase stage (Fig. 5h), anaphase with chromosomal loss (Fig. 5i), bridge in anaphase (Fig. 5j), anaphase/telophase with vagrant chromosome (Fig. 5k, n), polar slip at anaphase/telophase (Fig. 5l, o) and anaphase/telophase with lagging chromosomes (Fig. 5m, p) were observed in the cells of the treatment group (Groups II and IV) exposed to NaCl stress (Fig. 5).

It has been reported that micronucleus usually occurs due to spindle fiber damage or chromosome breaks/fragments that cause abnormal segregation of chromosomes in anaphase^55^. Bud formation is similar to MN formation and leads to loss of genetic material in both formations^56,57^. Lobed nuclei are formed as a result of inhibition of 214 DNA synthesis in the S phase of the interphase^58^. The presence of vagrant chromosomes causes the separation of different numbers of chromosomes into sister cells, followed by the formation of sister cells with unequal size or irregular nuclei in interphase^59^. It has been reported that bridges are formed by the breaking and recombination of chromosomes^60^. It has been determined that chromosomal stickiness occurs with the formation of subchromatid links between chromosomes as a result of entanglement of inter-chromosomal chromatin fibrils^61^. Lagging chromosomes resulted due to failure of the chromosomes to become attached to the spindle fiber and to move to either of the two poles^62^. It has been reported that malfunction of the mitotic spindle causes chromosomal loss^63^.

### The role of FA on the biochemical parameters

**Figure 6:**
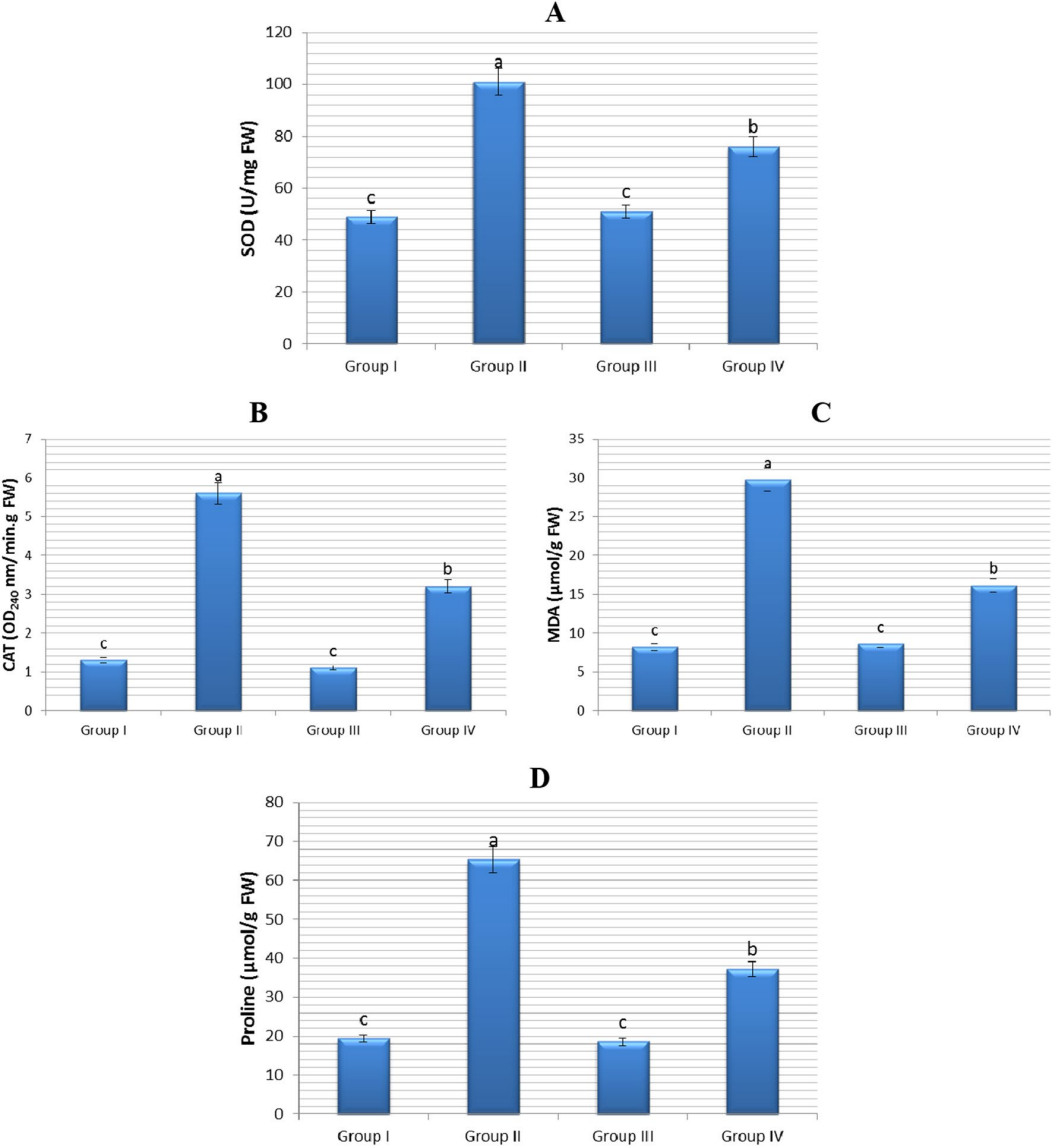
Impacts of exogenous FA administration on certain biochemical parameters of *Allium cepa* bulbs. (**A**) SOD (U/mg FW) (**B**) CAT (OD240 nm/min g FW) (**C**) MDA (µmol/g FW) (**D**) Proline (µmol/g FW). Tap water medium (control)/Group I, 0.15 M NaCl medium/Group II, 0.1 nM FA medium/Group III, 0.1 nM FA + 0.15 M NaCl medium/Group IV. Standard deviation (± SD) shown with error bars. Different letters^(a–c)^ indicate average *p* < 0.05 is important.

Salt stress leads to the formation of excessive amounts of reactive oxygen species (ROS). ROS, formed by the effect of salinity, disrupts the normal metabolism by causing lipid peroxidation, enzyme inhibition, protein and nucleic acid denaturation^64^. Plants possess antioxidant enzymes such as superoxide dismutase (SOD) and catalase (CAT) that act as ROS scavengers to alleviate oxidative damage caused by salt stress^65,66^. The effects of salt stress (0.15 M NaCl), FA (0.1 nM) and salt + FA (0.15 M NaCl + 0.1 nM FA) treatments on SOD and catalase CAT activity in the root cells of onion plant are given in Fig. 6. There was no statistically significant difference in SOD and CAT contents in the root cell of bulbs (Group III) treated with FA only compared to control (Group I) bulbs grown in tap water medium. The SOD and CAT contents in the root cells of Group I (control) bulbs were found to be 49 ± 2.5 U/mg FW and 1.3 ± 0.7 OD_240_ nm/min. g FW, respectively. In the root cells of Group III bulbs, these parameters were found to be 51 ± 2.7 U/mg FW and 1.1 ± 0.4 OD_240_ nm/min. g FW. This result revealed that exogenous FA administration did not cause an additional ROS generation in root cells.

But, SOD and CAT enzyme contents in the root cells of onion plant were markedly elevated by NaCl stress. In the root cells of Group I (control) bulbs grown in tap water medium, SOD and CAT contents were 49 ± 2.5 U/mg FW and 1.3 ± 0.7 OD240 nm/min. g FW, respectively; in the root cells of Group II bulbs grown in 0.15 M NaCl medium, these enzyme contents were 101 ± 5.0 U/mg FW and 5.6 ± 1.1 OD240 nm/min g FW, respectively. In other words, 0.15 M salt stress application significantly increased the SOD level in root cells of bulbs (Group II) by 106% and CAT level by 331% compared to control/Group I (Fig. 6). The increase in SOD and CAT contents in the roots of Group II seedlings is an important indicator of NaCl-induced ROS formation and oxidative stress. It has also been supported by many studies that antioxidant enzyme activities (SOD and CAT) increase in response to salt stress and salt tolerance is directly proportional to increased enzyme activities^67,68^.

On the other hand, application of 0.1 nM exogenous FA to Group IV bulbs grown in 0.15 M NaCl medium resulted in a significant decrease in SOD and CAT contents (p < 0.05). The root cells of Group II bulbs grown in 0.15 M NaCl medium had 101 ± 5.0 U/mg FW SOD activity and 5.6 ± 1.1 OD240 nm/min g FW CAT activity. In the root cells of FA-treated Group IV bulbs grown at this salt level, the activities of these enzymes were 76 ± 4.3 U/mg FW and 3.2 ± 0.8 OD240 nm/min g FW, respectively. That is, exogenous FA application (0.1 nM FA + 0.15 M NaCl) in onion (Group IV) plants under salt stress decreased SOD content by 25% and CAT content by 43%, when compared to salt-stressed plants alone/Grup II (Fig. 6). The decrease in antioxidant enzyme content in the roots of Group IV seedlings is an evidence that exogenous FA application contributes to the fight against ROS of the onion plant, relieving the effect of stress and increasing salt tolerance.

Membrane damage resulting from salinity is generally caused by membrane lipid peroxidation^69^. Determining the level of malondialdehyde (MDA), the end product of lipid peroxidation, determines the severity of oxidative stress, and it is a frequently used method to understand the sensitivity level of the plant^70^. The effects of salt stress (0.15 M NaCl), FA (0.1 nM) and salt + FA (0.15 M NaCl + 0.1 nM FA) treatments on MDA content in the root cells of onion plant are given in Fig. 6. There was no statistically significant difference in MDA contents in the root cells of bulbs (Group III) treated with FA only compared to control (Group I) bulbs grown in tap water medium. The MDA content in the root cells of Group I (control) bulbs was found to be 8.2 ± 2.4 µmol/g FW. In the root cells of Group III bulbs, this content was found to be 8.6 ± 2.1 µmol/g FW. This result revealed that exogenous FA administration did not cause membrane damage in root cells.

Whereas, MDA content in the root cells of onion plant was markedly elevated by NaCl stress. In the root cells of Group I (control) bulbs grown in tap water medium, MDA content was 8.2 ± 2.4 µmol/g FW; in the root cells of Group II bulbs grown in 0.15 M NaCl medium, this value was 29.7 ± 4.3 µmol/g FW. In other words, 0.15 M salt stress application significantly increased the MDA level in root cells of bulbs (Group II) by 3.6 folds compared to control/Group I (Fig. 6). The increase in MDA content in the root cells of Group II seedlings is a marker of oxidations in cell membranes a result of NaCl stress. It has also been reported in many studies that salt stress increases the amount of intrinsic MDA in plants by stimulating lipid peroxidation^71–73^.

On the other hand, application of 0.1 nM exogenous FA to Group IV bulbs grown in 0.15 M NaCl medium resulted in a significant decrease in MDA content. The root cells of Group II bulbs grown in 0.15 M NaCl medium had 29.7 ± 4.3 µmol/g FW MDA content. In the root cells of FA-treated Group IV bulbs grown at this salt level, the content of MDA was 16.1 ± 3.1 µmol/g FW. That is, exogenous FA application (0.1 nM FA + 0.15 M NaCl) in onion (Group IV) plants under salt stress decreased the MDA content by 1.8 folds (almost 46%), when compared to salt-stressed plants alone/Group II (Fig. 6). Exogenous FA application both alleviated the lipid peroxidation damage in the membranes and helped to increase tolerance to salinity by reducing the MDA content in the root cells of Group IV seedlings. Because MDA level did not increase significantly in genotypes with high salt tolerance, but increased significantly in sensitive genotypes^67^.

Accumulation of various amino acids is observed in plants exposed to salt stress. One of the most important of these amino acids is proline (PR). PR is an amino acid that plays an effective role in maintaining osmotic balance and protecting intracellular structures in plants exposed to stress^74,75^. The effects of salt stress (0.15 M NaCl), FA (0.1 nM) and salt + FA (0.15 M NaCl + 0.1 nM FA) treatments on free PR content in the root cells of onion plant are given in Fig. 6. There was no statistically significant difference in free PR contents in the root cells of bulbs (Group III) treated with FA only compared to control (Group I) bulbs grown in tap water medium. The free PR content in the root cells of Group I (control) bulbs was found to be 19.4 ± 2.5 µmol/g FW. In the root cells of Group III bulbs, this amino acid content was found to be 18.6 ± 2.2 µmol/g FW.

Whereas, free PR content in the root cells of onion plant was markedly elevated by NaCl stress. In the root cells of Group I (control) bulbs grown in tap water medium, free PR content was 19.4 ± 2.5 µmol/g FW; in the root cells of Group II bulbs grown in 0.15 M NaCl medium, this value was 65.3 ± 3.8 µmol/g FW. In other words, 0.15 M salt stress application significantly increased the free PR level in root cells of bulbs (Group II) by 3.4 times compared to control/Group I (Fig. 6). It has been reported in many studies that the PR amino acid accumulates in plant cells in large amounts under salt stress conditions^67,76^ and that there is a positive relationship between PR accumulation and stress tolerance^77,78^. In this study, a positive correlation was found between PR amino acid and accumulation of SOD, CAT and MDA. This finding shows that PR attenuates the effect of NaCl that causes degradation on cell membranes^79^, protects cell membranes^80^, and participates effectively in the clearance of produced ROS^81^ and Thus, it shows that it protects cells from oxidative damage.

On the other hand, application of 0.1 nM exogenous FA to Group IV bulbs grown in 0.15 M NaCl medium resulted in a significant decrease in free PR content. The root cells of Group II bulbs grown in 0.15 M NaCl medium had 65.3 ± 3.8 µmol/g FW free PR content. In the root cells of FA-treated Group IV bulbs grown at this salt level, free PR content was 37.2 ± 3.6 µmol/g FW. That is, exogenous FA application (0.1 nM FA + 0.15 M NaCl) in onion (Group IV) plants under salt stress decreased the PR content by approximately 1.8 times, when compared to salt-stressed plants alone/Group II (Fig. 6). The fact that exogenous FA application decreased free PR content in Group IV seedlings is an indication that salt stress damage is alleviated and salt tolerance is achieved.

This study is the first study in the literature on the effects of exogenous FA on SOD and CAT activities and MDA and free PR contents in root cells of plants under salt stress conditions. However, there are research findings that high doses of exogenous FA application increase the contents of SOD, CAT, MDA and free PR in leaf cells of various plants grown under stress-free conditions^18,43,82^, which is inconsistent with the current research results. Because, in the current study, it was determined that FA administered at low doses did not cause any change in the contents of SOD, CAT, MDA and free PR in the root cells of onion plant. As a result, FA may exhibit different effects on the mentioned parameters under unstressed conditions, depending on the plant species, plant organ and application dose.

### The role of FA on the anatomic parameters

**Figure 7:**
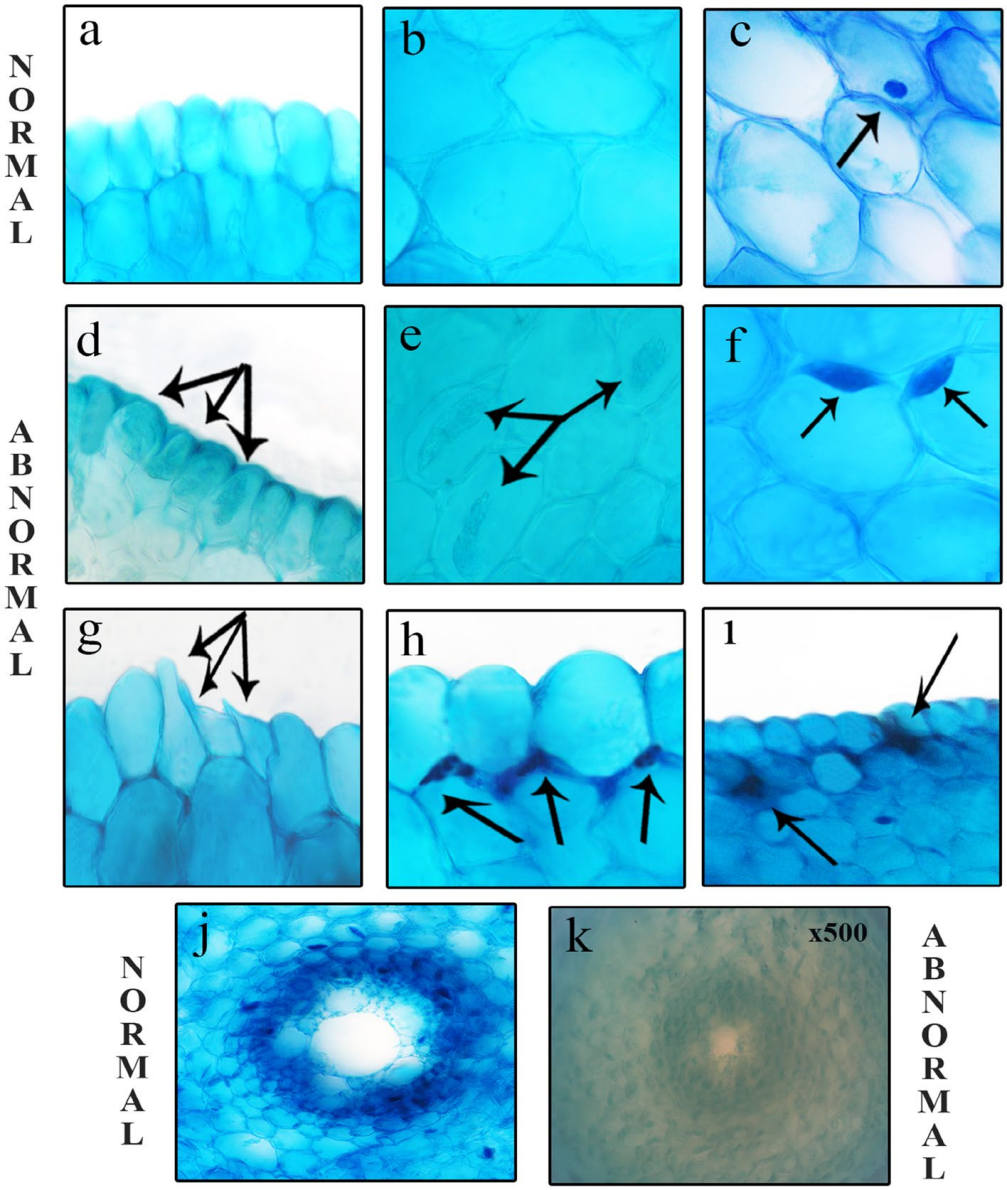
Meristematic cell injuries induced by NaCl (**a**) healthy appearance of epidermis cells (**b**) healthy appearance of cortex cells (**c**) healthy appearance of cell nucleus (oval) (**d**) accumulation of some chemical substances in epidermis cells *(arrows)* (**e**) accumulation of chemical substances in the cells of cortex layer *(arrows)* (**f**) flattened cells nuclei *(arrows)* (**g**) epidermal cell injury *(arrows)* (**h**) wall thickening in the cortex cells *(arrows)* **i**) necrotic areas *(arrows)* (**j**) normal appearance of transmission tissue (**k**) indistinct transmission tissue.

Roots are an important organ that connects the plant to the soil, takes water and nutrients and carries them to the above-ground organs, and secretes some hormones and organic compounds. The effects of salt stress (0.15 M NaCl), FA (0.1 nM) and salt + FA (0.15 M NaCl + 0.1 nM FA) treatments on the root anatomical structure of onion plant are given in Table 1 and Fig. 7.

**Table 1:** The degree of damage to the root anatomical structure of *Allium cepa* L.

Application groups	ACE	ACC	FCN	ECI	CWT	NA	ITT
Tap water/Group I/Control	−	−	−	−	−	−	−
0.15 M NaCl/Group II	+ +	+ +	+ +	+ +	+ +	+ +	+ +
0.1 nM FA/Group III	−	−	−	−	−	−	−
0.1 nM FA + 0.15 M NaCl/Group IV	+	+	+	+	+	+	+

Accumulation of some chemical substances in epidermis cells (ACE); accumulation of some chemical substances in cortex cells (ACC); fattened cell nucleus (FCN); epidermis cell injury (ECI); cortex cell wall thickening (CWT); necrotic areas (NA); indistinct transmission tissue (ITT). (−) no damage, ( +) little damage, (+ +) severe damage.

As a result of microscopic examination of the preparations prepared from root cross-sections of Group I/control bulbs grown in tap water medium and Group III bulbs grown in FA medium alone, it was determined that they had normal root anatomical structure and no damage or change occurred (Fig. 7). Similar results were obtained from the study of Diniz and Oliveira^83^, in which they reported that no change occurred in the root anatomical structure of *Zea mays* L. seedlings grown in media containing 0.1 mM and 0.2 mM FA.

A total of 7 types of abnormalities, including accumulation of chemical substances in the cells of epidermis and cortex layer (Fig. 7d, e), flattened cells nuclei (Fig. 7f), epidermal cell injury (Fig. 7g), wall thickening in the cortex cells (Fig. 7h), necrotic areas (Fig. 7i) and indistinct transmission tissue (Fig. 7k) were observed in the root cells of treatment group (Groups II and IV) exposed to NaCl stress (Fig. 7). All these damage and changes in the root anatomical structures of Group II and Group IV bulbs exposed to salt stress are the result of the defensive responses of cells and tissues to alleviate the effects of stress and increase salt tolerance.

One of the most important indicators that NaCl stress causes toxicity so severe that it disrupts the integrity of the cell wall can be epidermis cell damage. Flattened cell nuclei may occur as a result of rupture of cell membranes and oxidative stress-induced DNA damage^84^. Plants exposed to stress develop mechanisms such as slowing down the transmission of substances in order to reduce the destructive effect of stress, and as a result, the accumulation of various chemicals in the cells and thickening of the cell wall in order to reduce permeability^85,86^. With the help of these defense mechanisms, they can reduce the harmful effects of stress and increase tolerance^87^.

On the other hand, externally applied FA (0.1 nM FA + 0.15 M NaCl) to onion (Group IV) plants exposed to salt stress significantly reduced the severity of root anatomical structure damage (Table 1). This study is the first study in the literature on the effects of exogenous FA on the root anatomy of plants grown under saline conditions.

## Conclusion

As a result of this study, various morphological, cytogenetic, biochemical and anatomical parameters were examined and it was determined that FA, a mycotoxin, could alleviate the harmful effects of salt stress in the onion (*Allium cepa* L.) plant. Accordingly, it can be concluded that the economic losses caused by salt stress can be prevented by using FA applications at appropriate doses in agriculture. In addition, this study will shed light on salt tolerance studies in plants to be carried out in the future.

## Data Availability

The datasets used and/or analyzed during the current study are available from the corresponding author on reasonable request.
